# The Molecular Biology of Brain Metastasis

**DOI:** 10.1155/2012/723541

**Published:** 2012-03-08

**Authors:** Gazanfar Rahmathulla, Steven A. Toms, Robert J. Weil

**Affiliations:** ^1^The Burkhardt Brain Tumor & Neuro-Oncology Center, Cleveland Clinic, Desk S-7, 9500 Euclid Avenue, Cleveland, OH 44195, USA; ^2^Department of Neurosurgery, Neurological Institute, Cleveland Clinic, Cleveland, OH 44195, USA; ^3^Division of Neurosciences, Department of Neurosurgery, Geisinger Medical Center, Danville, PA 17822, USA

## Abstract

Metastasis to the central nervous system (CNS) remains a major cause of morbidity and mortality in patients with systemic cancers. Various crucial interactions between the brain environment and tumor cells take place during the development of the cancer at its new location. The rapid expansion in molecular biology and genetics has advanced our knowledge of the underlying mechanisms involved, from invasion to final colonization of new organ tissues. Understanding the various events occurring at each stage should enable targeted drug delivery and individualized treatments for patients, with better outcomes and fewer side effects. This paper summarizes the principal molecular and genetic mechanisms that underlie the development of brain metastasis (BrM).

## 1. Introduction

Brain metastases are the most frequently diagnosed intracranial neoplasms in adults, with an annual incidence estimated at 200,000 cases in the USA alone [1], an incidence 10 times greater than primary brain tumors [2]. Up to 20–40% of patients with adult systemic malignancies will develop brain metastases in the course of their disease; about 10–20% will be symptomatic [3, 4]. Improved treatment options for systemic disease, along with tools that permit less invasive screening, often when patients are asymptomatic, have increased patient survival, paradoxically escalating both its incidence and prevalence. A variety of systemic malignancies can metastasize to the central nervous system (CNS), although the majority of the lesions come from lung cancer (40–50%) followed by breast cancer (20–30%), melanoma (5–10%), lymphoma, and various other primary sites like the gastrointestinal tract (4–6%) and prostate [5, 6].

More than a century ago, Stephen Paget advanced his “seed and soil” hypothesis, which suggests that the occurrence of brain metastases is not random, but is secondary to certain tumor cells—“the seed”—having an attraction for the surrounding environment—“the soil” [7]. The hypothesis envisages three principles: first, that neoplasms are composed of heterogeneous subpopulations of cells, with different characteristics; second, that only a selectively “fit” subpopulation of cells will survive and multiply, invade, and migrate to other locations; finally, that colonization depends on tumor cell “seed” and host microenvironment “soil” interactions [8]. According to Ewing, circulatory patterns are responsible for the organ-specific spread between the primary tumor and their final destination [9]. Although complex, the metastatic process can be broadly divided into two main stages, the first being the migration of tumor cells from their primary tumor environment to various distant tissues and the second being the colonization of these tumor cells in their new location [10]. Underlying these two main stages are a number of cellular hallmarks taking place during the development and metastasis of human tumors [11]. The various molecular, genetic, and epigenetic changes that occur define the multistep dissemination process of the tumor, also known as the “metastatic cascade.”

Most BrMs occur in the cerebral hemispheres (80%), followed by the cerebellum (15%) and the brainstem (5%), corresponding to vascular distribution and tissue volumes [12]. BrMs are a major cause of morbidity and mortality, with clinical features of the metastasis corresponding to the location, causing focal neurological deficits, or presenting with nonspecific central nervous system features such as headache, cognitive impairment, and seizures [13]. The central nervous system (CNS) acts as a “safe haven,” generally beyond the reach of nearly all chemotherapeutic agents. The blood brain barrier (BBB) prevents the entry of most chemotherapeutic agents, and so the brain can act as a refuge for metastatic tumors [14]. The microenvironment of the CNS is exceptional in having a high chloride content, enabling tumors which prefer this environment, such as neuroepithelial tumors like small cell cancer of the lung and melanoma, to colonize, while potentially inhibiting invasion by other cancer cell types without this predilection [15]. Treatments targeting metastatic intracranial disease include surgery, whole-brain radiation therapy (WBRT), stereotactic radiosurgery (SRS), alone or in combination with various targeted agents, and generalized chemotherapy [16]. Following WBRT, survival ranges from anywhere between 4 and 6 months and can be as long as 24 months [17]. Various combinations of surgery, SRS, WBRT, and chemotherapy have been used to improve overall survival, obtain good clinical outcomes, and prevent recurrence of disease.

This paper will focus on metastatic brain tumors describing the hallmarks acquired in the metastatic cascade, which finally brings cancer cells to their “safe haven” in the CNS. The mechanisms through which cancer cells escape their primary focus of origin, invade adjacent tissues making their way into the microvasculature (intravasation), evade cell death, and make their way to a distant site (extravasation), finally proliferating and colonizing this new location, are outlined. With further understanding of the various molecular events that occur in metastasis, future-targeted therapies may lead to prevention or a slowdown in the development of BrM and more effective and less toxic therapy (ies).

## 2. The Metastatic Process

**Figure 1:**
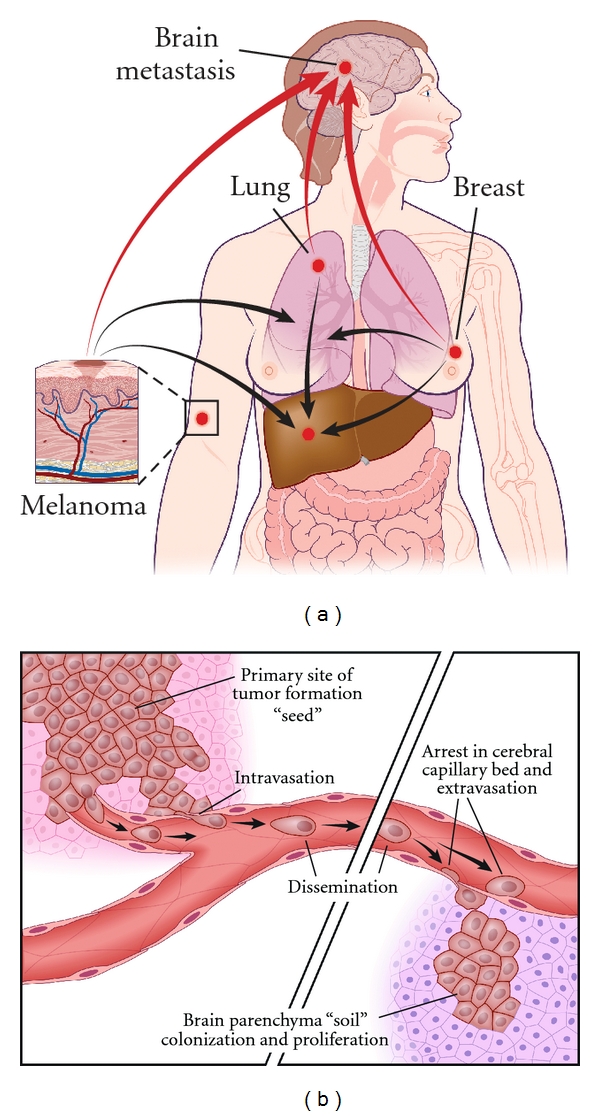
Schematics of the process of metastasis. (a) Formation of metastatic tumor cell lines at primary sites like breast, lung, and skin (melanoma) seen as the red nodes. Metastasis from these primary sites then spreads to the brain via the circulatory system (red arrows) and also to adjacent sites like the liver, bone, lung, and lymph nodes (black arrows). The inset shows the primary site of melanoma cells proliferating and migrating towards the vasculature, subsequently disseminating to secondary organ sites. (b) The metastatic tumor cells detach from the primary site and penetrate the adjacent parenchyma to reach the blood vessels. On reaching the vessels, the cells invade and enter the circulation (intravasation) and then disseminate within the vascular system (left half of figure). These cells eventually adhere to secondary sites “soil” to then extravasate out of the blood vessels and for colonies of metastatic cells (right half of figure).

The ability of cancer cells to sever their link to the primary tumor site and commence the metastatic process begins once specific functions have been acquired by an appropriate subset of cancer cells. The multistep cascade can be grouped into two stages: migration, which includes intravasation, dissemination, and extravasation, and colonization (Figures 1(a) and 1(b)). We will review below the underlying pathobiology within each stage.

### 2.1. Migration

#### 2.1.1. Cellular Heterogeneity and Proliferation

**Figure 2:**
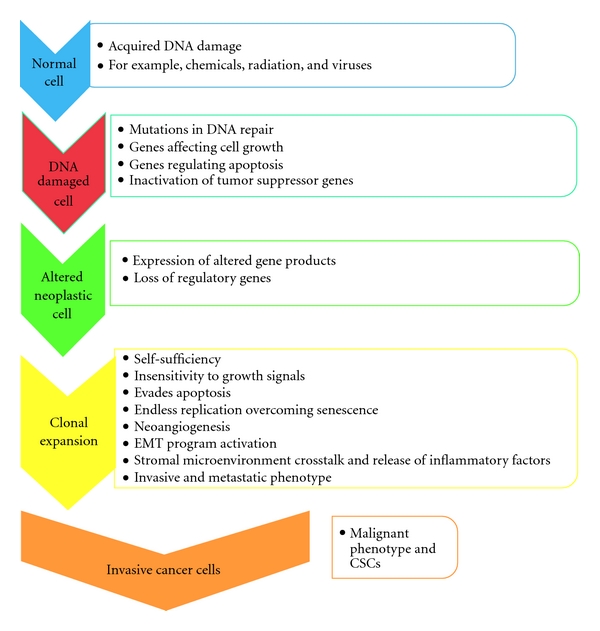
Schematic of the metastatic cascade. Cascade of events taking place at the primary site during oncogenesis, illustrating the steps creating the neoplastic cell line followed by clonal expansion and survival of the fittest cells, becoming the invasive and metastatic phenotype.

The primary tumor consists of cancer cells which are genetically heterogeneous and have varying potentials to metastasize. These include the cell's ability to invade adjacent tissues, initiate (neo-) angiogenesis, disseminate, and adhere to new tissue substrates, while expressing an affinity for the CNS [3, 18]. Tumor cells have the ability to evade the structural organization present in normal tissues and cells. In spite of being exposed to various environmental pressures such as hypoxia and nutrient deprivation, low pH, poor blood supply, and immune and inflammatory mediators, a subset of tumor cells survive these pressures with the ability to metastasize to distant sites. Additionally, tumor cells are able to evade growth suppressors, which limit cell growth and proliferation, as well as circumvent inhibitors of cell proliferation such as cell cycle checkpoint and DNA damage control systems. Tumor cells can also resist apoptosis (programmed cell death) by the increased expression of antiapoptotic regulators (Bcl-2, Bcl-x_L_), survival signals (lgf 1/2), and downregulating proapoptotic factors (Bax, Bim, and Puma) [19]. The primary tumor cells have the ability to acquire genetic and epigenetic mutations such as DNA methylation and histone modification, allowing the fittest group of cells to survive [10, 20]. Emerging evidence also suggests that microRNA (miRNA) species interactions with pseudogenes may modify gene expression in cancer [21]. Various genetic mutations result in the ability of tumor cells to commence the proliferative process, and a number of genes associated with this process are listed in Table 1. Clonal expansion of these surviving fit cells leads to an acquisition of further changes, making subsequent cell lines progressively more carcinogenic (Figure 2).

Observations within the primary tumor mass have revealed the presence of heterogeneous cell lines including cancer stem cells (CSCs), partially differentiated progenitor cells, and fully differentiated end-stage cells; these appear to recapitulate the same hierarchal patterns in normal tissue types but in an uncontrolled manner [22]. Present evidence suggests that these CSCs may be the primary drivers of the enhanced malignant potential of primary tumors, giving origin to their aggressive phenotypes with the ability to degrade the extracellular matrix (ECM), invade blood vessels and lymph nodes, migrate, extravasate, colonize, and renew themselves at their new locations [23, 24]. These CSCs can reside in clusters or niches, at two or more locations within the primary tumor cell mass [23, 24]. Thus, the key role a CSC plays in the metastatic cascade cannot be overstated, due to its ability to initiate tumor proliferation and “self-renew” itself at alternative tissue locations. Other observations reveal that, in addition to the abilities discussed, they are also motile and invasive and are resilient to the apoptotic process [25]. 

#### 2.1.2. Epithelial-Mesenchymal Transition (EMT)

 The epithelial-mesenchymal transition (EMT), which is currently at the forefront of investigation by numerous groups, describes a temporary, reversible phenomenon wherein cells can dedifferentiate, migrate to a distant focus, and then redifferentiate back to their original cell, forming a new structure [26]. Signals activating the EMT can be intrinsic, such as gene mutations, and extrinsic, such as growth factor signaling. Transdifferentiation appears to be initiated by release of certain EMT-inducing transcription factors (EMT-TFs) that transform epithelial cells into mesenchymal derivatives, giving these cells the capacity to invade, resist apoptosis, and disseminate [11, 27]. Transforming growth factor *β* (TGF*β*) [28], hepatocyte growth factor (HGF) [29], epidermal growth factor (EGF), insulin-like growth factor (IGF) [30], fibroblast growth factor (FGF), and members of the Notch signaling family [31] play a role in inducing the EMT pathway. More recent evidence indicates that the EMT program enables non-CSCs to derive characteristics of the CSC state, which enables them to invade and disseminate from the primary tumor to a distant, metastatic focus [32]. Some of these traits include the ability to loosen adherent junctions, express matrix-degrading enzymes, resist apoptosis, and to undergo morphological conversion. Using the EMT program, cancer cells can, transiently or for longer time frames, activate themselves and acquire attributes critical to survival and dissemination. To activate the EMT, a certain amount of crosstalk has to exist between the tumor cells and adjacent stromal cells, which are done by various EMT-TFs and signals from within the adjacent tumor stroma [11, 32].

#### 2.1.3. Interactions with Tumor Stroma

 Progression in cancer also involves activating a number of cells in the adjacent stroma via paracrine signaling [33]. These stromal cells, including endothelial cells, pericytes, fibroblasts, and leukocytes, provide a number of protumorigenic factors which sustain tumor growth. The two prominent cell types are the cancer-associated fibroblasts (CAFs) and the pericytes [34]. These cells produce growth factors, hormones, and cytokines that promote tumor proliferation. CAFs are known to express high amounts of TGF*β*, HGF, EGF, FGF, canonical Wnt families, and cytokines like stromal-derived factor- (SDF-) 1*α* (CXL12) and interleukin-6 (IL-6) [35]. Invasion of cancer cells can be enhanced by stromal macrophages which supply matrix-degrading enzymes such as matrix metalloproteinases and cysteine cathepsin protease [36]. Experimental tumor models suggest that cancer cells release factors such as CSF-1, which stimulates macrophages in the tumor microenvironment, with the subsequent release of EGF, which promotes proliferation of the tumor mass [37]. In addition, CAFs are activated by various inflammatory mediators and induced to produce increased quantities of VEGF, FGF-2, among other cytokines and growth factors, which recruits endothelial progenitor cells thereby promoting angiogenesis [38, 39]. This dynamic stromal environment further stresses the tumor cells, potentially enhancing additional genomic instability, and heterogeneity and epigenetic dysregulation [40]. 

#### 2.1.4. Local Invasion

 Once the phenotypically aggressive clone has developed, spread of the tumor consists of a series of two sequential steps: namely, invasion of the extracellular matrix (ECM), with penetration into the vasculature and hematogenous dissemination to the CNS. Tumor expansion causes adjacent ECM compression and modifies lymphatic and blood vessel flow, eventually leading to basement membrane (BM) thinning. Combined with the various molecular and cellular events, this leads to eventual tumor metastasis.

 To reach the circulation, tumor cells must penetrate the BM, traverse the extracellular connective tissue matrix (ECM) tissue, and then breach the vascular basement membrane (VBM) to enter the circulation. The process is dependent on a number of protein complexes that regulate cellular interactions and proteolytic enzymes, with degradation of the ECM, which permits extravasation.

#### 2.1.5. E-Cadherin-Catenin Complex (ECCC), Integrins, and Other Molecules

 The E-cadherin-catenin molecular complex is essential to maintain a normal and tumoral cytoarchitecture. It is a necessary mediator of cell-cell adhesion that, among other functions, determines the polarity of normal (and tumor) cells and their organization into tissues [41]. Cadherin molecules are integral cell membrane glycoproteins that interact in a homophilic manner with one another. They have a stable extracellular fragment and possess a cytoplasmic undercoat protein of one or more proteins called catenins. In the process of tumor metastasis, tumor clones become discohesive, fail to adhere to one another, and develop a more disordered cytoarchitecture, which allows these cells to separate from the tumor mass. E-cadherin maintains cell adhesion by anchoring its cytoplasmic domain to actin cytoskeleton via *α*-catenin and *β*-catenin. Infiltrating malignancies have mutations in the genes for *α*- and *β*-catenins and E-cadherin, thus decreasing the expression of this complex. This has been correlated with invasion, metastasis, and an unfavorable prognosis. Furthermore, DNA hypermethylation of the promoter region of *E-cadherin* can diminish or silence its expression, thereby disturbing ECCC function, and is a common event in many metastatic cancers [42]. N-cadherin is another molecule connected to the cellular cytoskeleton via *α*-catenin and *β*-catenin in a manner similar to E-cadherin. One of the hallmarks of the EMT described above is a cadherin switch, with loss of epithelial E-cadherin and gain of mesenchymal N-cadherin functions. This induces loss of epithelial cellular affinity, while at the same time increasing the affinity of cells for the mesenchymal cells like fibroblasts. Gain-of-function mutations in *N-cadherin* also trigger increased migration and invasion in tumors [43].

*Integrins* are another family of major adhesion and signaling receptor proteins linking the ECM to the cellular actin cytoskeletal structure called focal adhesions and play an important role in mediating cell migration and invasion [44]. They trigger a variety of signal transduction pathways and regulate cytoskeletal organization, specific gene expression, control of growth, and apoptosis. Animal models of human nonsmall cell lung cancer (NSCLC) have shown that blocking *α*_3_ *β*_1_ integrin significantly decreases brain metastasis [45]. Additionally, Carbonell et al. have shown that blocking the *β*_1_ integrin subunit prevents adhesion to the VBM and attenuates the development of metastasis [46]. Integrins induce the release of a key mediator in signaling known as focal adhesion kinase (FAK). FAK is a ubiquitously expressed nonreceptor cytoplasmic tyrosine kinase, thought to play a key role in migration and proliferation, by providing abnormal signals for survival, EMT, invasion, and angiogenesis [47]. FAK may also play an important role in the regulation of CSCs. Dephosphorylation and inhibition of FAK at the Y397 locus via the activated Ras (rat sarcoma) oncogene promote tumor migration by facilitating focal adhesion at the leading edge of tumor cells [15, 48].

 The ability of tumor cells to escape the primary site is dependent on their ability to remodel the ECM. This remodeling occurs by breaking down or degrading the ECM via proteolytic enzymes, thus creating a pathway for invasion. The advancing edge of tumor cells posses the ability to carry out this proteolytic activity by releasing signals that promote cell proliferation and angiogenesis in the metastatic cascade. Neurotrophins (NTs) promote brain invasion by enhancing the production of heparinase, which is an ECM proteolytic enzyme. Heparinase is a *β*-d-glucuronidase that cleaves the heparin sulfate chain of the ECM. It is the prominent heparin sulfate degradative enzyme [49] and is known to destroy both the ECM and the BBB [3]. Evidence suggests the presence of NTs at the tumor-brain interface in melanomas, and reports have suggested a role for the p75 NT receptor in brain metastasis [50].

*Matrix metalloproteinases (MMPs)* are members of a family of zinc-dependent endopeptidases that function at physiological pH and help remodeling human connective tissue at low levels. About 25 human family members have been identified, and they have been grouped according to their substrate on which they act, namely, collagenases, stromelysins, matrilysins, and gelatinases [51]. They also play a critical role in the EMT and tumor microenvironment [52]. Cytokines and inducers present on the surface of tumor cells in the ECM regulate their expression. Once these MMPs are induced and stimulated, they aid in breakdown of type I collagen, fibronectin, and laminin in the ECM [53] and enhance tumor cell migration. MMP activity correlates with invasiveness, metastasis, and poor prognosis [54]. In one study of brain metastasis, MMP-2 was identified in all metastases regardless of site of origin. Moreover, MMP-2 activity correlated inversely with survival [55]. In a murine tumor model, the incidence of brain metastasis was reduced by 75% when compared to the wild type following the use of tissue inhibitor of metalloprotease1 (TIMP-1), which suggests that inhibitors of MMPs suppress BrMs [56].

*The urokinase-type plasminogen activator (uPA)* system consists of uPA, its receptor (uPAR), and plasminogen. The uPA binds to the receptor uPA-R (CD87), the activity of which is regulated by the action of plasminogen activator inhibitor type 1 and 2 (PAI-1/2) on the cell membrane and causes urokinase to convert plasminogen to plasmin. The proteolytic activity of plasmin then degrades components of the ECM including fibrin, fibronectin, proteoglycans, and laminin. Further, plasmin activates other proteolytic enzymes with resultant local invasion and migration [57]. As far back as 1994, researchers have found that there is a high level of uPA in metastatic tumors, that uPA correlates with necrosis and edema, and that there is an inverse correlation with a tumor's levels of uPA and survival [58]. Additionally, high levels of uPA and absent tissue plasminogen activator (tPA) correlate with aggressiveness and decreased survival [58].

More recent evidence describes the role of  “invadopodia,” which are three-dimensional protrusive processes, compared to the two-dimensional lamellipodia and filopodia, in metastatic invasion [59]. Invadopodia appear to share a number of structural and functional features with filopodia, but spatially focus proteolytic secretion, remodeling the ECM matrix and establishing tracts supporting subsequent invasion [60]. Integrins play a major role in organizing the components, triggering the formation of invadopodia. *α*_3_ *β*_1_ activation promotes Src-dependent tyrosine phosphorylation of p190RhoGAP, via RhoGTPases family, which activates invadopodia and invasion [61]. Integrins also appear to focus proteolytic activity to the region of these processes, as in melanoma cells, where collagen-induced *α*_3_ *β*_1_ association with the serine protease Seprase (surface-expressed protease) enhances the activity of matrix-degrading enzymes focally at the invadopodia [62]. Numerous cancer cell lines such as melanoma, breast cancer, glioma, and head and neck cancer have shown the presence of invadopodia. A number of other molecules, such as EGF, HGF, or TGF-*β*, can induce their formation as well [63]. The release of tumor-released chemokines such as CSF-1 and PIGF attract tumor-associated macrophages (TAM) to the microenvironment, which in turn release multiple factors stimulating invadopodia [64]. In addition, a family of proteins called aquaporins may also facilitate migration. Aquaporin-dependent tumor angiogenesis and metastases enhance water transport in the lamellipodia of migrating cells [65]. Studies on brain-specific breast metastasis reveal that increased expression of *KCNMA1*, a gene encoding for a big conductance type potassium channel (BKCa) that is upregulated in breast cancer, leads to greater invasiveness and transendothelial migration [66].

#### 2.1.6. Genetic Alterations

 Several known tumor suppressor genes (TSGs) that function at the level of escape and migration/intravasation are worth exploring and are enumerated in Table 2. The best known of these is the *KiSS1* gene on chromosome 1. *KiSS1* encodes metastin, which is a ligand of the orphan G protein couples receptor hOT7T175. Lee et al. [67] have found that the forced expression of *KiSS1* suppressed both melanoma and breast metastasis. Other authors have found an inverse correlation between *KiSS1* expression and melanoma progression [68].

*KAI1 (CD82),* a TSG on chromosome 11p11.2, regulates adhesion, migration, growth, and differentiation of tumor cell lines. *KAI1* expression is inversely correlated with prostate cancer progression [69] as well as breast [26, 27] and melanoma metastasis [28]. Additionally, *KAI1* is known to be associated with the epidermal growth factor receptor (EGFR), discussed later in this paper, and is thought to affect the Rho GTPase pathway [29] resulting in suppression of lamellipodia formation and migration [30].

Hypermethylation of the TSG *Drg1* inhibits both liver metastasis and colorectal carcinoma invasion [70]. Conversely, overexpression of *Drg1* has been linked to resistance to irinotecan chemotherapy [71]. Finally, in a murine model of breast cancer metastasis, the Notch signaling pathway was found to be activated via increased Jag2 mRNA levels, thereby, creating a cell line that was both more migratory and more invasive in collagen assays. Additionally, inactivation of the Notch pathway significantly decreased tumor cell migratory and invasive activity [72]. In addition to the suppressor genes responsible for invasion and metastasis, there are a number of promoter genes responsible for invasion and metastasis as well, a few of which are enumerated in Table 3. Genetic activation or inactivation of promoter/suppressor genes in human cancer can be the result of mutations, deletions, loss of heterozygosity, multiplication, and translocation [73]. The same genes that are responsible for normal cellular functioning, signaling, signal transduction, modulating, and mediating cellular response are frequently the genes that enhance invasion and metastasis when altered by genetic or epigenetic dysfunction [74, 75].

**Figure 3:**
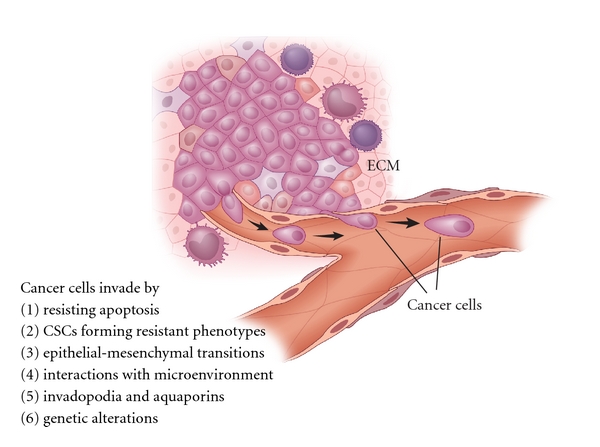
Invasion and migration. Subsets of cancer cells at the primary site develop an invasive phenotype; survive environmental pressures such as hypoxia and nutrient deprivation, low pH, poor blood supply, immune, and inflammatory mediators, gaining the ability to metastasize to distant sites. These cancer cells can evade growth suppressors and circumvent inhibitors of cell proliferation to intravasate and disseminate to various other sites.

These changes within the primary tumor microenvironment give rise to an “active seed” ready to implant itself in a fertile environmental “soil” (Figure 3). These cellular modifications enable the next steps of migration, namely, dissemination and extravasation.

#### 2.1.7. Dissemination

Once a cancer cell has breached its microenvironment and arrived at the vasculature (brain metastasis) or lymphatic system (other sites), the tumor cell must survive its exposure to high shear forces and varied stress patterns. Tumor cells respond by reenforcing their cytoskeleton and increasing the ability to adhere to the vascular wall [76]. More recent experimental evidence suggests shear induces a paradoxical enhancement of adhesion to the VBM via activation of Src [77] and FAK phosphorylation seen in colon cancer cell lines [78]. On adhering to endothelium of target tissue, the tumor cells behave like macrophages, creating pseudopodia, and penetrating the cell-cell junctions, driven by dynamic remodeling of the cellular cytoskeleton [60]. There are a subset of circulating tumor cells which maintain their physical plasticity and, although much larger in diameter (20–30 *μ*) than lung capillaries (~8 *μ*), can survive the sieving action of lung capillaries. These cells can be found either growing as clumps in the lung or colonizing other organ sites [10]. Cancer cells in circulation appear to attract platelets because of their expressed surface tissue proteins, and these protect the cells from the immune system [79]. Once these mobile cancer cells get lodged in a secondary organ tissue site, there are two pathways for colonization. One is mediated by cellular diapedesis, extravasation, and proliferation of the tumor cell mass, whereas the other consists of accumulation of tumor cells within the site of obstruction in the foreign tissue vascular bed, wherein they proliferate, prior to their rupture into the adjacent stroma where they begin to grow [80].

### 2.2. Colonization

#### 2.2.1. Organ-Specific Infiltration

**Figure 4:**
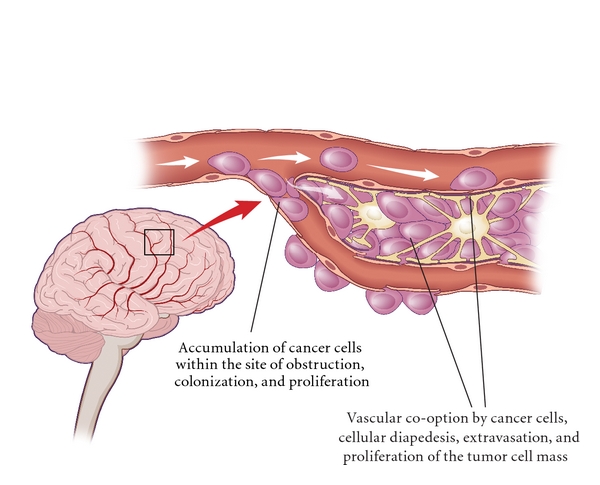
Colonization of metastatic tumor cells in the brain. Overexpression of the adhesion molecules makes it easy for tumor cells to target and adhere to endothelial lining in the parenchyma, making it possible for these endothelial-adhesive interactions to enhance the possibility of brain metastasis. Direct neurotropic interactions with brain homing mechanisms result in BrM. “Vascular co-option” is the ability of metastatic cells to grow along the preexisting vessels, and once adherent to the VBM, tumor cells can extravasate into the parenchyma, the VBM thus being the “soil” for BrM.

 Subsequent to intravasation and dissemination, special mechanisms are necessary to extravasate and colonize secondary sites. The metastatic deposits occur in certain organ tissues because of the influence of hematogenous dynamics, for example, colon cancer metastasis preferentially metastasizing to the liver because of mesenteric circulation and large vascular sinusoids [81]. The overexpression of the cell adhesion molecule, metadherin, in breast cancer makes it easier for tumor cells to target and adhere to endothelial lining in the lung parenchyma [82], making it possible for these endothelial-adhesive interactions to enhance the possibility of brain metastasis. Although the exact causes of preferential metastatic sites have not been clearly elucidated, one theory states that direct neurotropic interactions with yet undiscovered brain homing mechanisms result in BrM. “Vascular co-option,” a term put forward by Carbonell et al., describes the ability of metastatic cells to grow along the preexisting vessels much before overt secondaries are detected. Once adherent to the VBM, tumor cells can extravasate into the parenchyma, the VBM thus being the “soil” for BrM (Figure 4) [46]. Saito et al. demonstrated that the pia-glial membrane along the external surface of blood vessels serves as a scaffold for the angiocentric spread of metastatic cells [83].

In a mouse model of CNS metastasis, tumor cells function like macrophages within the vasculature and during extravasation, expressing CD11b, Iba1, F4/80, CD68, CD45, and CXCR, which are proteins normally expressed specifically by macrophages [84]. The ability of tumor cells to mimic macrophages may enable them to evade the immune system while in the vasculature.

#### 2.2.2. The BBB, Function of the Brain Microenvironment, and Brain Metastasis

Passage of tumor cells across the BBB occurs via mechanisms which have not yet been delineated fully. Recently, three proteins that mediate breast metastasis to the brain and lungs have been described, namely, cyclooxygenase 2 or COX2 (also known as PTGS2), EGFR, ligand and heparin binding epidermal growth factor (HBEGF). These proteins facilitate extravasation through nonfenestrated blood vessels and enhance colonization [85]. Other molecules targeting organ specific colonization may also be expressed by the cancer cell [86]. These molecules include ezrin (an intracellular protein necessary for the survival of osteosarcoma cells in the lung) and serine-threonine kinase 11 (STK11, or LKB1, a metastasis suppressor which regulates *NEDD9* in lung cancer) [87]. 

#### 2.2.3. Neoangiogenesis and Proliferation

A key component of both primary and secondary (metastatic) tumor growth at any site is angiogenesis [8]. Experimental systems, using breast or melanoma cell lines to model BrM, have revealed that growth may occur by utilizing preexisting vasculature, or co-opting these vessels rather than inducing new vessel formation (neoangiogenesis) [46, 88, 89]. Kusters et al. [90], using a melanoma cell line in a murine metastatic brain tumor model, showed that growth of the metastatic tumor up to 3 mm could occur without inducing the angiogenic switch [91]. Carbonell et al. have also shown that *β*_1_ integrin, expressed by the metastatic tumor cell line, is the key molecule to co-opt adjacent blood vessels to the growing tumor.

Various angiogenic factors have been scrutinized as viable targets for treatment. Vascular endothelia growth factor (VEGF) is the most commonly recognized angiogenic factor. VEGF expression in breast cancer plays a role in metastasis and inhibition with a tyrosine kinase receptor inhibitor-reduced growth and angiogenesis [92]. *SSecks* (Src-suppressed C kinase substrate) has been observed to decrease VEGF expression. This protein also stimulates proangiogenic angiopoietin 1 and regulates bran angiogenesis and tight junction creation, thus helping to regulate BBB differentiation [93].

MMP-9/gelatinase B complex, a member of the MMP family, and PAI-1, a uPA cell surface receptor, may play roles in angiogenesis [94]. The role in angiogenesis and uniqueness of Plexin D1 expression was explored in tumor cells and vasculogenesis. Neoplastic cells expressed Plexin D1 as well as tumor vasculature, while its expression in nonneoplastic tissue was restricted to a small subset of activated macrophages, which suggests that Plexin D1 may play a significant role in tumor angiogenesis [95]. Overexpression of hexokinase 2 (HK2), which plays a key role in glucose metabolism and apoptosis, may also influence BrM in breast and other cancers. Researchers at the National Cancer Institute found that both mRNA and protein levels of HK2 are elevated in brain metastatic derivative cell lines compared to the parental cell line *in vitro. *Knockdown of expression reduced cell proliferation, which implies that HK2 contributes to the proliferation and growth of breast cancer metastasis. Finally, increased expression of HK2 is associated with poor survival after craniotomy [96, 97].

At least two tumor suppressor genes that function at the proliferation level of the metastatic cascade have been described. The first, *NM23,* regulates cell growth by encoding for a nucleotide diphosphate protein kinase that interacts with menin, a TSG encoded by *MEN1 *[98]. NM23 is thought to reduce signal transduction and thereby decrease anchorage independent colonization, invasion, and motility [99]. In melanoma, decreased expression is correlated with increased brain metastasis [100]. Another tumor suppressor gene, *BrMS1,* located at 11q13 is altered in many melanomas and breast cancers. BrMS1 prevents disseminated tumor cell growth by restoring the normal gap junction phenotype and maintaining cell-to-cell communication in the primary tumor [101]. Seraj et al. [102] found an inverse correlation between the expression of BrMS1 and the metastatic potential in melanoma.

#### 2.2.4. Cascade-Nonspecific Contributors to Metastasis

 There are certain molecular contributions that cannot be attributed to a specific step in the cascade, either because they are active at every level or, as in most cases, their true function is yet to be discovered (see Tables 1 and 2). These molecular entities are on the forefront of cancer research and are worth addressing. *Zeb-1*, the zinc finger E-box homeobox transcription factor, is overexpressed in metastatic cancers. This overexpression leads to epithelial-mesenchymal transition and increased metastasis. Mutation of *Zeb-1* leads to decreases in the proliferation of progenitor cells in mutant mice. This mutation may be a target for metastatic prevention at the progenitor level [103].

Several other genetic markers have been located that pertain to metastasis in particular. A deletion of the 4q arm in lung (both small and nonsmall cell) metastasis to the brain and bone has been documented [104]. Additionally, in NSCLC, overexpression of *CDH2* (N-cadherin), *KIFC1*, and *FALZ *is highly predictive of metastasis to the brain in early and advanced lung cancer. Therefore, these genes may be used to predict a high risk of metastasis early in the diagnosis [105]. In prostate cancer, increased expression of *KLF6-SV1,* the Kruppel-like factor tumor suppressor gene, predicts poorer survival and is correlated with increased metastasis to the lymphatic system, the brain, and bone [106]. Finally, overexpression of homeoprotein Six-1, a transcriptional regulator, increased TGF-*β* signaling and metastasis in breast cancer with significantly shortened relapse times [107]. Gaining a better understanding of the role(s) of these genes and others will be important to deeper knowledge of the metastatic cascade.

#### 2.2.5. Overview of microRNAs (miRNAs) and Their Emerging Role in Oncogenesis

 Recent evidence has established an important role of microRNAs in cell and tissue development, proliferation and motility via their ability to repress mRNA translation or induce mRNA degradation [108]. The dysregulated expression of a single miRNA can cause a cascade of silencing events capable of eliciting disease development in humans, which includes cancer [109]. Breast cancer is found to possess aberrant regulation of several miRNAs [110]. They also play a prominent role in expression of EMT-related genes. Finally, pseudogenes, which encode RNAs that do not have to produce proteins but can compete for microRNA binding, may play a role in tumorigenesis and metastasis. Poliseno et al. [111]. Recently described the functional relationship between the mRNAs produced by the *PTEN* tumor suppressor gene and its pseudogene *PTENP1. PTENP1* regulates cellular levels of PTEN and can exert a growth-suppressive role and the *PTENP1* locus is lost in several human tumors, including prostate and colon cancer. They also showed that other cancer-related genes possess pseudogenes, including oncogenic *KRAS*. While the role of miRNAs and psuedogenes in metastasis is beyond the scope of this summary, several recent, excellent reports detail this emerging field [21, 111, 112].

## 3. Conclusion

The metastatic cascade, from its initiation to its completion in the brain, is an extremely complex, multistep process. For patients, the progression in the metastatic cascade to brain colonization is becoming both an increasingly treatable and yet simultaneously and increasingly prevalent feature of their disease, with consequent morbidity. As more evidence regarding the molecular and genetic factors that contribute to the cascade appears, targeting this ominous disease with multiple therapeutic strategies comes closer.

Knowledge of the metastatic process may lead to better detection and treatment of brain metastases. The goal however will be to utilize all the information gained at the genetic and molecular level to stop cancer, at the primary proliferative stage, preventing the initiation of the metastatic cascade and subsequent development of brain metastasis.

## Figures and Tables

**Table 1:** Genes associated with increased metastatic potential.

Genes	Cancer site (primary)	Role and implications	OMIM no.	Chromosome location
*RHoC*	Melanoma	Regulates remodeling of actin cytoskeleton during morphogenesis and motility. Important in tumor cell invasion	165380	1p21-p13

*LOX*	Breast Head and neck cancer	Increases invasiveness of hypoxic human cancer cells through cell matrix adhesion and focal adhesion kinase activity	153455	5q23.1-q23.2

*VEGF*	Lung Breast Melanoma Colon	Angiogenic growth factor Inhibition decreases brain metastasis formation; reduces blood vessel formation and cell proliferation; increases apoptosis	192240	6p21.1

*CSF1*	Breast Lung	Stimulate macrophage proliferation and subsequent release of growth factors	120420	1p13.3

*ID1*	Breast Lung	Involved in matrix remodeling, intracellular signaling, and angiogenesis	600349	20q11.21

*TWIST1*	Breast Gastric Rhabdomyosarcoma Melanoma Hepatocellular	Causes loss of E-cadherin-mediated cell-cell adhesion, activates mesenchymal markers, and induces cell motility by promoting epithelial-mesenchymal transition	601622	7p21.1

*MET*	Renal cell cancer	Affects a wide range of biological activity depending on the cell target, varying from mitogenesis, morphogenesis, and motogenesis	164860	7q31.2

*MMP-9*	Colorectal Breast Melanoma Chondrosarcoma	Extracellular matrix degradation, tissue remodeling	120361	20q13.12

*NEDD9*	Melanoma	Acquisition of a metastatic potential	602265	6p24.2

*LEF1*	Lung	Transcriptional effecter—WNT pathway; predilection for brain metastasis Knockdown inhibits brain metastasis, decreases colony formation;* in vitro decreases invasion *	153254	4q25

*HOXB9*	Lung Breast	Homeobox gene family; critical for embryonic segmentation and patterning. Also a TCF4 target Knockdown *in vitro* decreased invasion and colony formation; *in vivo* appears to inhibit brain metastasis	142964	17q21.32

*BMP4*	Lung Colorectal	Plays an essential role in embryonic development and may be an essential component of the epithelial-mesenchymal transition	112262	14q22.2

*STAT3*	Melanoma	Cell signaling transcription factor Reduction suppresses brain metastasis; decreases angiogenesis *in vivo* and cellular invasion *in vitro *	102582	17q21.2

**Table 2:** Representative metastasis and invasion suppressor genes.

Gene	Cancer/metastatic tumor	Function(s) of protein	OMIM no.	Chromosome Location
*NM23*	Breast, colon, melanoma	A histidine kinase. Nm23 phosphorylates KSR and can lead to decreased ERK 1/2 activation. appears to play a role in normal development and differentiation	156490	17q21.3

*MKK4*	Breast, ovarian, and prostate	A mitogen-activated protein kinase (MAPKK) that phosphorylates p38 and Jun (JNK) kinases	601335	17p11.2

*BRMS1*	Breast, melanoma	Functions in gap-junction communication	606259	11q13.1-q13.2

*KiSS1*	Breast, melanoma	A G-protein coupled receptor ligand, also known as metastin.	603286	1q32

*KAI1 (CD82)*	Bladder, breast, lung, pancreas, and prostate	Interact with beta-catenin-reptin and histone deacetylases. It may desensitize EGFR activity, also known as kangai	600623	11p11.2

*CD44*	Breast, colon, lung, melanoma, prostate	An integral cell membrane glycoprotein that affects cell adhesion. Decreased expression due in part to hypermethylation	107269	11pter-p13

*CRSP3*	Melanoma	A transcriptional coactivator that may work through the enhancer binding factor Sp1	605042	

*RHOGDI2*	Bladder, breast, colon, kidney, liver, lung, and prostate	Regulates function of Rho and Rac, GTP-binding proteins of the Ras superfamily		11p11.2

*VDUP1*	Melanoma	A differentiation factor via thioredoxin inhibition	606599	1q21

*PTEN/MMAC1*	Breast, colon, endometrial, germ cell, kidney, lung, melanoma, and thyroid	A homologue of cytoskeletal tension, leading to invasion and metastasis through interaction with actin filaments at focal adhesions	601728	10q23.31

*VHL*	Renal cell, pheochromocytoma, and hemangioblastoma	Encodes protein products playing an essential role in microtubule stability, orientation, tumor suppression, cilia formation, signaling of cytokines, and extracellular matrix assembly	608537	3p25.3

*TIMP2*	Melanoma	Protease inhibitor plays a role in preventing excessive ECM disruption	188825	17q25.3

*SMAD4*	Pancreatic cancer, colorectal, and prostate	Transcription factor, pivotal role in signal transduction of TGF*β*	600993	18q21.2

*RRM1*	Lung	Cell cycle regulator	180410	11p15.4

*PTPN11*	Lung, colon, thyroid, and melanoma	Regulates tyrosine phosphatase, proliferation, differentiation, motility, and apoptosis of cells	176876	12q24.1

*CDH1*	Gastric, breast	Cellular adherens junctional protein	192090	16q22.1

*CASP8*	Gastric, breast, lung, and PNETs	Apoptotic cascade via aspartate-specific cysteine proteases	601763	2q33

Definitions: EGFR: epidermal growth factor; ERK: extracellular signal-regulated kinase; JNK: Jun-terminal kinase; KSR: kinase suppressor of Ras. OMIM no.: Online Mendelian Inheritance in Man Identification number (http://www.ncbi.nlm.nih.gov/), which provides detailed information and references for these genes, their protein products, and potential functions.

**Table 3:** Representative metastasis and invasion promoter genes.

Gene	Cancer/metastatic tumor	Function(s) of protein	OMIM no.	Chromosome location
*ERBB2 (HER2)*	Breast	Receptor tyrosine kinase, critical component of IL6, and cytokine signaling	164870	17q21.1

*TIAM1*	Lymphomas, renal cell cancer, colon, prostate, and breast	Activates Rho-like GTPase Rac1, Tiam1Rac1 signaling which affects invasion in numerous ways	600687	21q22.1

*SRC*	Colorectal, breast, melanoma, and lung	Critical role in cellular signal transduction pathways, regulating cell division, motility, adhesion, angiogenesis, and survival	190090	20q12-q13

*S100A4*	Colorectal Breast Gastric cancers	Increases endothelial cell motility and induces angiogenesis, increases invasive properties through deregulation of the extracellular matrix	114210	1q21

*MTA1*	Breast Ovary Lung Gastrointestinal Colorectal	Nucleosome remodeling and deacetylating (NuRD) complex serves multiple functions in cellular signaling, chromosome remodeling and transcription processes, that are important in the progression, invasion, and growth of metastatic epithelial cells	603526	14q32.3

*KRAS*	Pancreatic Lung Colorectal	Encode GDP/GTP-binding proteins involved in signal transduction during cellular proliferation, differentiation, and senescence	190070	12p12.1

*HRAS*	Bladder Renal Thyroid	Small GTPase growth promoting factor	190020	11p15.5

## Abbreviations

AKAP12: A-kinase anchor protein 12
ARHGDIB(RhoGD12): Rho GDP dissociation inhibitor beta
BBB: Blood brain barrier
BKCa: Big conductance type potassium channel
BrMS1: Breast cancer metastasis suppressor 1
CASP8: Caspase8
CDH1: Cadherin 1
CD11b: Cluster of differentiation molecule 11b
CD45: Cluster of differentiation 45
CD44: cluster of differentiation 44
CD6: Cluster of differentiation 68
CDH2: Neutral cadherin (N-cadherin), when overexpressed with FALZ and KlFC1 genes) predicts metastasis to brain
COX2: Cyclooxygenase 2 (also known as PTGS2)
CRSP3: Cofactor required for Sp1 transcriptional activation subunit 3
CXCR: Chemokine receptor
CD87: uPA binds to the receptor uPA-R
CNS: Central nervous system
CXCL12/CXCR4: Chemokine/receptor system
CXCR7: Alternate receptor to CXCL12
CSF-1: Colony stimulating factor-1
Drg-1: Differentiation-related, putative metastatic suppressor gene
ECM: Extracellular matrix
EGFR: Epidermal growth factor receptor
ERBB2(HER2): Avian erythroblastic leukemia homolog 2
F4/80: Transmembrane protein present on cell surface of mouse
FALZ: Overexpression with CDH2 and KlFC1 genes predicts metastasis to the brain
FAK: Focal adhesion kinase
HBEGF: Heparin binding epidermal growth factor
HSP27: Heat shock protein
HK2: Hexokinase 2
HOXB9: homeobox B9
HRAS: Harvey rat sarcoma viral oncogene homolog
Iba1: Ionized calcium binding adaptor molecule 1
KAI1 (CD82) gene: Tumor suppressor gene
KCNMA1: tumor suppressor gene
KIFC1: Overexpression with CDH2 and FALZ genes predicts metastasis to brain
KISS-1: Gene that encodes metastin
KLF6-SV1: Kruppel-like factor tumor suppressor gene
KRAS: Kirsten rat sarcoma viral oncogene homolog
LEF1: Lymphoid enhancing factor 1
MIB-1: Mindbomb homolog 1 labeling index
MMAC1: Multiple advanced cancers
MMPs: Matrix metalloprotease
MEN1: Multiple endocrine neoplasia type 1
MTA1: Metastasis-associated protein 1
NM23(NME1): Metastasis suppressor gene also known as NDP kinase
NSCLC: Nonsmall cell lung cancer
NTs: Neurotrophins
PAI-1/2: plasminogen activator inhibitor type 1 and 2
PTEN: Chromosome 10
PIGF: Phosphatidylinositol glycan, class F
PTPN11: Protein-tyrosine phosphatase, nonreceptor type 11
PTEN: Phosphatase and tensin homologue deleted on chromosome 10
Ras: Oncogene (rat sarcoma) gene
RRM1: Ribonucleotide reductase, M12 subunit
sHSP: Small heat shock protein
SSecks: Src-suppressed C kinase substrate
Six-1: Homeoprotein transcriptional regulator
ST6GALNAC5: *α*2,6-sialyltransferase
S100A4: S100 calcium-binding protein A4
SMAD4: Sma- and Ma-related protein 4
SRC: Rous sarcoma virus protein
TIAM1: T-cell lymphoma invasion and metastasis 1
TIMP2: Tissue inhibitor of metalloproteinase 2
TCF: T-cell factor pathway
TGF: Transforming growth factor
TIMP-1: Tissue inhibitor of metalloprotease 1
tPA: Tissue plasminogen activator
uPA: Urokinase-type plasminogen activator
uPA-R (CD87): Urokinase-type plasminogen activator receptor
VBM: Vascular basement membrane
VEGF: Vascular endothelial growth factor
VDUP: Vitamin D3-upregulated protein
VHL: Von Hippel-Lindau tumor suppressor
WNT: Wingless integration gene
Zeb-1: Zinc finger E-box-binding homeobox 1
OMIM no.: Online Mendelian Inheritance in Man Identification Number (http://www.ncbi.nlm.nih.gov/), which provides detailed information and references for these genes, their protein products, and potential functions.
